# Functional Assessment of Population and Tumor-Associated APE1 Protein Variants

**DOI:** 10.1371/journal.pone.0065922

**Published:** 2013-06-11

**Authors:** Jennifer L. Illuzzi, Nicole A. Harris, Brittney A. Manvilla, Daemyung Kim, Mengxia Li, Alexander C. Drohat, David M. Wilson

**Affiliations:** 1 Laboratory of Molecular Gerontology, National Institute on Aging, National Institutes of Health, Baltimore, Maryland, United States of America; 2 Department of Cardiopathology, Sanford Burnham Medical Research Institute, Orlando, Florida, United States of America; 3 Department of Biochemistry and Molecular Biology, University of Maryland School of Medicine, Baltimore, Maryland, United States of America; 4 Marlene and Stewart Greenebaum Cancer Center, University of Maryland School of Medicine, Baltimore, Maryland, United States of America; 5 Department of Genetic Engineering, Cheongju University, Cheongju, Republic of Korea; University of Medicine and Dentistry of New Jersey, United States of America

## Abstract

Apurinic/apyrimidinic endonuclease 1 (APE1) is the predominant AP site repair enzyme in mammals. APE1 also maintains 3′–5′ exonuclease and 3′-repair activities, and regulates transcription factor DNA binding through its REF-1 function. Since complete or severe APE1 deficiency leads to embryonic lethality and cell death, it has been hypothesized that APE1 protein variants with slightly impaired function will contribute to disease etiology. Our data indicate that except for the endometrial cancer-associated APE1 variant R237C, the polymorphic variants Q51H, I64V and D148E, the rare population variants G241R, P311S and A317V, and the tumor-associated variant P112L exhibit normal thermodynamic stability of protein folding; abasic endonuclease, 3′–5′ exonuclease and REF-1 activities; coordination during the early steps of base excision repair; and intracellular distribution when expressed exogenously in HeLa cells. The R237C mutant displayed reduced AP-DNA complex stability, 3′–5′ exonuclease activity and 3′-damage processing. Re-sequencing of the exonic regions of *APE1* uncovered no novel amino acid substitutions in the 60 cancer cell lines of the NCI-60 panel, or in HeLa or T98G cancer cell lines; only the common D148E and Q51H variants were observed. Our results indicate that *APE1* missense mutations are seemingly rare and that the cancer-associated R237C variant may represent a reduced-function susceptibility allele.

## Introduction

Cellular DNA is susceptible to continual damage from endogenous sources, such as oxygen radicals produced by mitochondrial respiration, from spontaneous decay due to the intrinsic instability of nucleic acids, and from a range of exogenous chemical and physical agents, including sunlight and ionizing radiation [1]. Abasic sites (apurinic/apyrimidinic or AP sites), which lack the instructional information found in the base moiety, are common lesions with the potential to be cytotoxic or mutagenic if left unrepaired [2]. AP sites are formed (i) as products of DNA glycosylases, which excise base lesions generated via deamination, oxidation or alkylation as part of base excision repair (BER), or (ii) through the spontaneous or damage-induced hydrolysis of the N-glycosylic bond that links the base moiety to the sugar phosphate DNA backbone. AP sites are corrected by AP endonucleases, which initiate nucleoside removal via strand incision as a central step in the BER pathway [3]–[5].

The major (if not sole) AP endonuclease in mammalian cells is APE1, which shares sequence and structural similarity to AP endonucleases in the *E.coli* exonuclease III (*xth*) family [6]–[8]. The human *APE1* gene is located on chromosome 14q11-12, spans ∼2.6 kb, and encodes a protein of 318 amino acids with a predicted molecular mass of ∼35 kDa. APE1 is a globular α/β protein that exhibits multiple functions in DNA repair and transcriptional regulation. For example, besides being the primary AP endonuclease in mammals, APE1 acts as a 3′-phophodiesterase to remove fragmented sugar moieties (*e.g.*, α,β-unsaturated aldehyde) found at DNA strand break ends [9]. APE1 has also been shown to have 3′-phosphatase, 3′–5′ exonuclease, RNase H and RNA cleavage activities [10]–[14]. Additionally, the N-terminal ∼65 residues of APE1, which are mostly missing from the bacterial counterpart, contribute to its so-called REF-1 function [15], [16]. In this capacity, APE1 acts as a reducing donor for oxidant-sensitive cysteines in certain transcription factors (*e.g.*, AP-1, p53 and NF-κB), thereby activating cognate DNA binding activity and modulating gene expression [15], [17].

Mice engineered to lack a central participant in the BER pathway (*e.g.*, APE1, the major gap-filling DNA polymerase, POLβ, or the single-strand break scaffold protein, XRCC1) are embryonic or post-natal lethal [18], [19]. This inviability likely stems from the accumulation of endogenous DNA damage during embryogenesis, resulting in aborted development. Since a severe reduction in the core components of BER appears to be incompatible with life, it has been hypothesized that mild reductions in BER capacity will be associated with disease risk, possibly in a relevant exposure-dependent manner [5]. Seemingly consistent with this notion, BER haploinsufficiency has been associated with reduced life expectancy, increased spontaneous mutagenesis and carcinogenesis, and genotoxin-dependent cancer susceptibility [20]–[23]. Notably, several amino acid variants have been identified in human BER proteins that potentially represent reduced-function alleles [5]. In addition, recent work of the Sweasy laboratory has found that tumor-associated variants of POLβ can induce cellular transformation and thus may contribute directly to the carcinogenic process [20]. In the study here, we characterized the functional and structural consequences of both population and cancer-associated amino acid variants observed in APE1.

## Materials and Methods

### APE1 Plasmid Constructs

Human APE1 variant expression constructs (Q51H, I64V, P112L, D148E, R237C, G241R, P311S, and A317V) were created using the QuikChange II Site-Directed Mutagenesis Kit (Stratagene). Forward and reverse primers containing the relevant nucleotide change (Table S1 in File S1) were generated following the mutagenic primer design guidelines of the manufacturer. To obtain duplex DNAs that harbor the specified nucleotide/amino acid change, the appropriate primer pair (125 ng of each oligonucleotide) was mixed with 10 ng of wild-type pET11a-APE1 plasmid [24], dNTPs and PfuUltra HF DNA polymerase, and subjected to the basic thermal cycling protocol outlined by the manufacturer. After digestion with Dpn1, the DNA mix was transformed into XL10-Gold Ultracompetent cells (Stratagene), and selected clones were sequence confirmed by the Johns Hopkins Synthesis and Sequencing Facility (Baltimore, MD).

To create the fluorescently-tagged APE1 variant expression systems, the APE1 cDNA was PCR amplified from the respective pET11a plasmid constructs above using the following primers: Forward, 5′-CCCCAAGCTTTAATGCCGAAGCGTGG-3′ and Reverse, 5′-CGGGATCCTCACAGTGCTAGGTATAGG-3′. The A317V variant was amplified using a modified reverse primer, which harbored the unique codon sequence for amino acid residue 317∶5′-CGGGATCCTTCACAG TACTAGGTATAGG-3′. The PCR products were digested with BamHI and HindIII (New England BioLabs) and subcloned into the pmCherry-C1 vector (Clontech). Recombinant plasmid sequences were confirmed as above.

### APE1 Protein Purification

pET11a expression plasmids containing either wild-type APE1 or an APE1 variant cDNA (see above) were transformed into BL21 (λDE3) bacterial cells (Novagen). Bacterial cells were grown at 37°C to an absorbance of 0.8 at OD_600_. IPTG was then added to a final concentration of 1 mM, and cells were grown overnight at 20°C. Bacteria were harvested and lysed by sonication, and the recombinant APE1 protein was purified from the clarified extracts using UNO Q12 and UNO S6 columns (BioRad) as described in Erzberger et al [24].

### Equilibrium Denaturation

Samples of APE1 or variants (0.40 uM) were incubated for 20 minutes at room temperature in buffer (0.02 M HEPES pH 7.5, 0.1 M NaCl, 0.2 mM EDTA, 2.5 mM MgCl_2_) that contained varying concentrations of guanidine HCl (GdnHCl) (0–6 M). The extent of protein unfolding was determined by measuring the wavelength for maximal protein fluorescence (λ_max_) from emission spectra collected with a QM-4 spectrofluorometer (Photon Technology International). The excitation wavelength was set at 295 nm to excite tryptophan but not tyrosine residues, and the excitation and emission slit widths were 0.5 mm and 1.25 mm, respectively. Three scans were used to determine an average value of λ_max_ for each sample. Chemical-induced protein denaturation was considered as a two-state transition between native (N) and unfolded (U) conformations giving distinct maximal emission wavelengths, λ_max_^N^ and λ_max_^U^. A control reaction showed that GdnHCl-induced unfolding of APE1 is reversible (data not shown). Following a linear extrapolation method [25], [26], the thermodynamic parameters of chemical-induced unfolding were determined by fitting the dependence of λ_max_ on [GdnHCl] to eq. 1, using non-linear regression with Grafit5 [27].
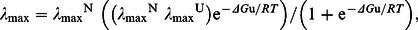
(1)where Δ*G*_u_ = Δ*G*_uw_–*m*_eq_[GdnHCl]. Δ*G*_u_ represents the free energy of protein denaturation at each concentration of GdnHCl. Δ*G*_uw_ is the extrapolated free energy of protein unfolding in the absence of denaturant, and *m*_eq_ relates the sensitivity of Δ*G*_u_ to the concentration of GdnHCl.

### Nuclease Assays

To measure AP endonuclease activity, 30 pg wild-type or variant APE1 protein was mixed with 0.5 pmol 5′-[^32^P]-labeled duplex 18F NMR DNA in 10 µl reactions containing 50 mM Tris pH 7.5, 25 mM NaCl, 1 mM MgCl_2_, 1 mM DTT and 0.01% Tween-20. Reactions were incubated at 37°C for 10 min. To inactivate the enzyme, an equal volume of stop buffer (90% formamide, 20 mM EDTA) was added and the mixture was heated at 95°C for 10 min. Following separation on a 20% polyacrylamide-urea denaturing gel, substrate and product bands were visualized by a Typoon Trio+ Variable Model Imager (Amersham Bioscience/GE Healthcare, Piscataway, NJ), and the signals were quantified using ImageQuant software (Molecular Dynamics, GE Healthcare). Enzyme kinetics were determined by running assays at substrate concentrations of 10, 25, 50, 100 and 200 nM, and plotting the results on Lineweaver-Burk graphs. The 3′ to 5′ exonuclease assays were carried out as above, except that 250 ng APE1 protein and 0.5 pmol 5′-[^32^P]-labeled partially duplex 15P or 17P DNA substrate were used. Reactions were stopped and resolved on an 18% polyacrylamide denaturing sequencing gel, before being analyzed as above. APE1 3′-repair activity was assessed against a 3′-phosphate-containing oligonucleotide duplex (15-p/34G; identical to 15P/34G, except 15-p possesses a 3′-phosphate group) as described in Wilson et al [14]. In brief, APE1 protein (1 ng) was incubated in 10 µl reactions consisting of 50 mM HEPES (pH 7.5), 20 mM KCl, 10 mM MgCl_2_, 2 mM DTT and 1 pmol 5′-[^32^P]-labeled DNA substrate. Samples were incubated for 10 min at 37°C and then processed as above. All statistical comparisons were carried out using the Student’s T-test.

### AP-DNA Binding Assay

The DNA binding assay was originally described in Wilson et al [28]. APE1 protein (2 ng) was incubated in 10 µl reactions containing 50 mM HEPES-KOH, pH 7.5, 50 mM KCl, 10% glycerol, 0.01% Triton X-100, 100 µg/mL BSA, and 4 mM EDTA with 100 fmol 18F NMR DNA for 5 min on ice. DNA binding reactions (10 µL) were resolved on a non-denaturing 8% polyacrylamide gel. Bound and unbound DNA was visualized and quantified as above. Statistical analysis was carried out using the Student’s T-test.

### REF-1 (Redox) Assay

EMSAs were performed as described [29] with minor modifications. In brief, 2.5 µg/µL APE1 protein was reduced with 1.0 mM DTT at 37°C for 10 min in 10 µl and then diluted 5-fold with PBS buffer to yield a final concentration of 0.5 µg/µL APE1 and 0.2 mM DTT. Nuclear extracts from HCT116 cells (see below) were treated with 0.01 mM diamide for 10 min at room temperature. To assess REF-1 activity, 1 µg of reduced APE1 protein was mixed with 6 µg of diamide-treated nuclear extract in EMSA buffer (10 mM Tris, pH 7.5, 50 mM NaCl, 1 mM MgCl_2_, 1 mM EDTA, 5% glycerol) in a volume of 18 µL and incubated for 30 min at room temperature. One µl of poly(dT-dA)-poly(dA-dT) was added and incubated for 5 min at room temperature. Next, 0.1 pmol of the ^32^P-labeled oligonucleotide duplex containing the AP-1 consensus binding sequence (5′-CGCTTGATGACTCAGCCGGAA-3′) was added, and the mixture was incubated for 30 min at room temperature; for controls, a mutant AP-1 consensus sequence was used (5′-CGCTTGATGACTTGGCCGGAA-3′). Samples were resolved on an 8% polyacrylamide nondenaturing gel in 0.5X TBE, and bound and unbound DNA was visualized and quantified as above.

### Nuclear and Cytoplasmic Extract Preparation

Cytoplasmic and nuclear extracts were prepared from human cells (HCT116 or HeLa) using the NE-PER kit from Thermo Scientific. In brief, cells were harvested from a confluent T-75 flask and processed using CER I (100 µL) and CER II (5.5 µL) solutions as described by the manufacturer. Following centrifugation at 16,000×g for 5 min, the supernatant (cytoplasmic extract) was retained. NER solution (50 µL) was added to the insoluble pellet, processed according to the manufacturer specifications, and following centrifugation at 14,000×g for 10 min, the supernatant (nuclear extract) was saved. Protein concentrations were quantified using the BCA protein assay kit (Bio-Rad), and extracts were stored at −80°C until needed.

### Reconstituted Repair Assay

Purified UDG (125 pg; New England Biolabs), APE1 (250 pg) and POLβ (41.5 pg; [30]) were mixed with a 34 base pair uracil-containing 5′-[^32^P]-labeled DNA substrate (1 pmol) and 100 µM dCTP in 10 µl containing 50 mM Tris-HCl, pH 8.0, 10 mM MgCl_2_, 0.01% Tween-20, 1 mM DTT. Reactions were incubated for 10 min at 37°C, stopped as described above, and resolved on a 15% polyacrylamide denaturing sequencing gel. Imaging and analysis were performed as stated ealier.

### Intracellular Localization

HeLa cells were grown in high glucose Dulbecco’s modified eagle’s media (DMEM, Invitrogen) supplemented with 10% fetal bovine serum (FBS, Invitrogen), 100 U/mL penicillin and 100 U/mL streptomycin (Invitrogen) at 37°C with 5% humidity. Cells were plated overnight, and the next day, cells were transfected using 0.25 µg of the designated pmCherry vector (see above) for every 1 µL of Lipofectamine 2000 (Invitrogen) in OPTIMEM media (Invitrogen). The cells were incubated for 4 hr at 37°C, and afterwards, the transfection media was aspirated and fresh DMEM media with FBS was added. The cells were incubated for an additional 20 hr at 37°C prior to visualization. Slides were prepared by washing the cells with 1X PBS and adding ProLong Gold plus DAPI (Molecular Probes) before sealing. Images were captured using a Zeiss Axiovert 200 M microscope with AxioCam Hrm and processed using the AxioVision software (Zeiss).

### Subcellular Fractionation

After transfection with an APE1 pmCherry vector (see above), cytoplasmic and nuclear extracts were prepared using the NE-PER extraction reagents above. Protein samples (25 µg) were separated on a 10% polyacrylamide-SDS gel and transferred to a polyvinylidene fluoride membrane. The membrane was blocked with 5% (w/v) milk in 1X Tris-buffered saline (TBS) containing 0.1% (w/v) Tween-20 (TBST), and probed overnight at 4°C using an in-house rabbit polyclonal primary antibody against APE1 (1∶1000 dilution). After three TBST washes, the membrane was incubated with secondary antibody (1∶1000, anti-rabbit, goat-HRP, Pierce) at room temperature for 1 hr, and subsequently washed, visualized with the SuperSignal West Femto Chemiluminescent kit (Thermo Scientific), and imaged on a ChemiDoc XRS^+^ (Bio-Rad).

### *APE1* Re-sequencing

Amplification of the *APE1* exons was performed by mixing 200 ng genomic DNA (obtained from the National Cancer Institute), 250 nM of each of the paired amplification primers (Table S2 in File S1), 1 mM dNTPs, and 1 µL Herculase II Fusion DNA polymerase in 1X Herculase II reaction buffer (Agilent Technologies). Cycling conditions were as follows: 5 min at 95°C; 35 cycles of 30 sec at 95°C, 45 sec at 61°C (59°C for Exon 5 product), and 1 min at 72°C; and 7 min at 72°C. The PCR products were sequenced at Johns Hopkins Synthesis and Sequencing Laboratory, using a primer binding platform within the amplification primers. Sequencing results for each exonic PCR product were submitted to BLAST analysis, and any nucleotide changes from transcript variant 3 of APE1 (NM_080649.1) were recorded (raw sequencing data available upon request).

## Results

### Background on APE1 Variants

**Figure 1:**
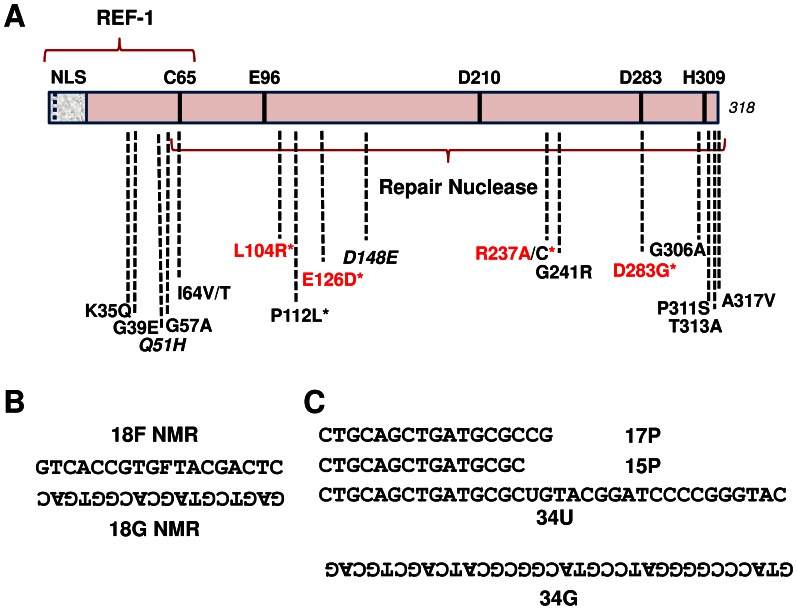
APE1 protein variants and oligonucleotide substrates. (**A**) Linear schematic of the 318 residue APE1 protein, including several reported amino acid substitutions. NLS = nuclear localization sequence; REF-1 = redox regulatory portion of the protein; italics = polymorphic variants; * = unique disease-associated variants; red text = variants with reduced AP endonuclease activity. The repair nuclease domain and several functionally important amino acids (C65, E96, D210, D283 and H309) are indicated. See Table 1 for additional details. (**B**) The 18F NMR and 18G NMR oligonucleotides were used to design the double-stranded AP endonuclease substrate. (**C**) The 15P or 17P oligonucleotide was annealed to the 34G oligonucleotide to generate a 3′-recessed exonuclease/repair substrate, whereas the 34U oligonucleotide was annealed to 34G to create the uracil-containing duplex for the reconstitution assay. Oligonucleotides are written 5′ to 3′, with the non-labeled strands written upside-down. F = the AP site analog, tetrahydrofuran.

Many re-sequencing efforts have been conducted over the past several years and have found that DNA repair genes, both in the normal healthy population and within disease samples, exhibit significant nucleotide sequence variability [31], [32]. In some instances, the single nucleotide polymorphism (SNP) or mutation alters the coding sequence of the gene, resulting in a protein with a variant amino acid composition. Over a decade ago, we characterized the functionality of several APE1 variants identified by us, or reported in the NCBI database or in published literature [33]. Since then, additional APE1 non-synonymous nucleotide substitutions have been deposited in the NCBI database, and in one study, were observed uniquely in endometrial cancer [34]. Figure 1A shows several reported APE1 variants at the start of this project. Table 1 lists the APE1 variants of focus for this paper, as well as their source, frequency of observation, and past functional characterization if available.

**Table 1:** APE1 variants and predicted impact of amino acid change.

APE1 Variant	Source	% Frequency	Functional Consequence	SIFT	CupSat		PolyPhen
					Overall Stability	Torsion	
Q51H	NCBI rs1048945	4.5	–	Possible AffectedFunction	Destabilizing	Unfavorable	Possible damaging
I64V	NCBI rs2307486	4.8	–	Tolerated	Destabilizing	Favorable	Resistant or no change
P112L	Tumor	Once	–	Tolerated	Destabilizing	Favorable	Resistant or no change
D148E	NCBI rs1130409	48.5	Normal AP endo*	Tolerated	Destabilizing	Favorable	Resistant or no change
R237C	Tumor	Once	–	Affected Function	Stabilizing	Unfavorable	Probably damaging
G241R	NCBI rs33956927	1.1	Normal (or increased) AP endo*	Tolerated	Stabilizing	Favorable	Resistant or no change
P311S	NCBI rs1803120	N/A	–	Affected Function	Stabilizing	Favorable	Probably damaging
A317V	NCBI rs1803118	N/A	–	Tolerated	Destabilizing	Unfavorable	Resistant or no change

Sorting intolerant from tolerant (SIFT) uses sequence homology to predict effects on protein function (http://sift.jcvi.org/). Scores <0.05 are considered deleterious, whereas those that are >0.5 are considered to be tolerated. The program polymorphic phenotypes (PolyPhen) predicts impact based on a set of empirical rules that apply to the protein’s sequence, phylogenetic and structural information (http://genetics.bwh.harvard.edu/pph/). Cologne University protein stability analysis tool (CupSat) predicts protein stability (http://cupsat.tu-bs.de/), and was employed using the PDB APE1 protein structure (1DE8).

* Described previously in [33]. N/A = not available. Once = it was observed a single time.

### Computational Assessment of APE1 Variant Structure/Function

As an initial assessment of the potential impact on protein structure/function, homology-based models of the APE1 variants (Table 1) were built with SWISS-MODEL using the wild-type APE1 crystal structure (PDB ID 1DE8) as a template. Superimposition of the variant and wild-type APE1 protein models revealed no major deviations in the amino acid backbone, secondary structure, or active site composition (Figure S1 in File S1). Three different on-line predictive modeling programs were also employed to assess whether the specific amino acid change might affect APE1 function (summarized in Table 1). The SIFT method, which is based on the degree of conservation of a particular amino acid within an alignment of closely related sequences, predicted that the Q51H variation would “possibly” affect protein function and that the R237C and P311S substitutions would affect protein function. CupSat, which estimates protein stability based on the nature of a specific amino acid change within a known structural environment, predicted that five of the mutations – Q51H, I64V, P112L, D148E and A317V – would be overall “destabilizing”, while Q51H, R237C and A317V would exhibit “unfavorable” torsion angle potentials. The third program, PolyPhen, which is based on straightforward empirical rules that are applied to the primary sequence, phylogenetic conservation and 3-D structural information, predicted that the Q51H variation was “possibly damaging” and that the R237C and P311S mutations were “probably damaging”. While the predictions from the three modeling programs were often different, all predicted that the Q51H change is likely to be damaging to APE1 structure/function; two of the programs predicted that the R237C and P311S substitutions will have a negative impact on structure/function. Consistent with the modeling predictions, previous studies found that D148E and G241R (which were considered to be non-damaging in all methods) exhibited essentially normal AP endonuclease activity *in vitro* [33]. The other substitutions, I64V, P112L and A317V, were predicted to have an adverse impact on APE1 structure/function only by the CupSat program. It is worth pointing out that none of the residue changes examined herein reside in a known post-translational modification site within APE1 [35].

### General Properties and Thermodynamic Stability of Recombinant APE1 Variants

**Figure 2:**
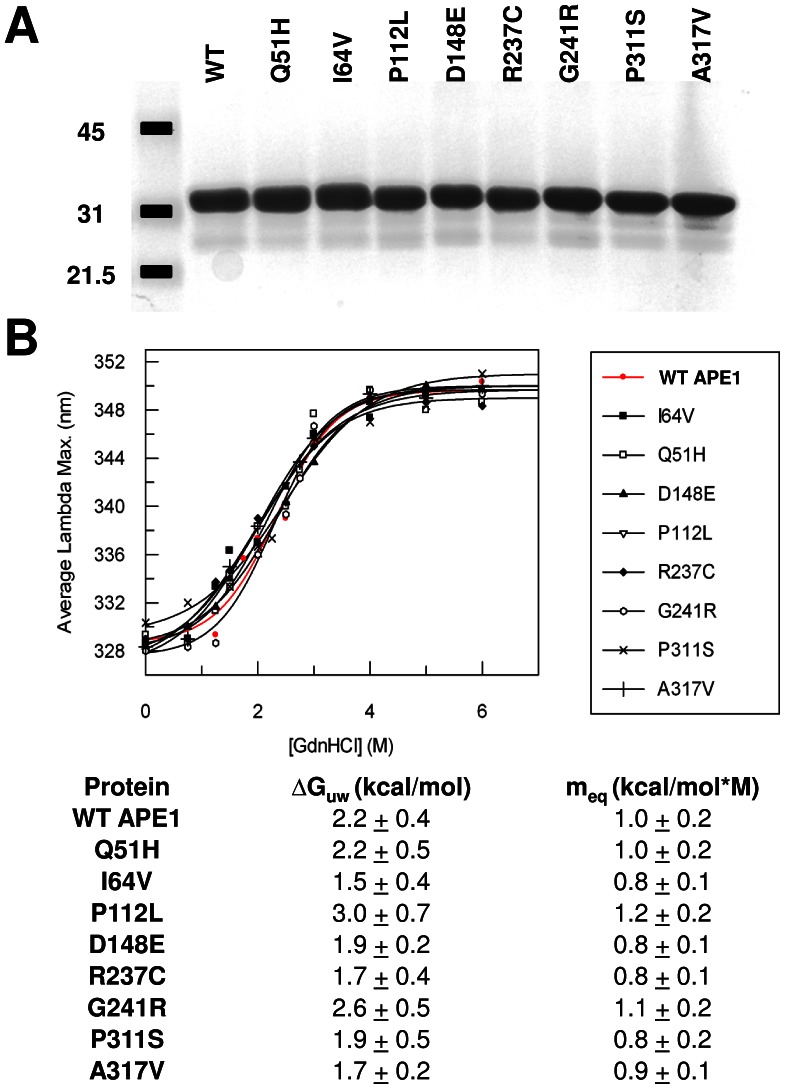
Variant proteins and thermodynamic stability of folding as determined by chemical denaturation. (**A**) Following purification, wild-type (WT) and variant APE1 proteins were quantified and analyzed (1 µg) by SDS-polyacrylamide gel electrophoresis and Coomassie blue staining. Shown is a representative gel image. Molecular mass standards are indicated to the left in kDa. (**B**) Profile of maximum wavelength emission for tryptophan fluorescence relative to GdnHCl concentration for the wild-type (WT) and variant APE1 proteins (top). The free energies of protein unfolding in the absence of denaturant (ΔG_uw_) and the values reflecting the dependence of the free energy on denaturant concentration (m_eq_) are shown (bottom).

The wild-type and variant APE1 proteins were found to exhibit similar expression levels in bacteria, solubility after cell sonication, and column elution profiles during purification, suggesting no major structural deformity or protein instability as a result of the amino acid change. Following isolation, each protein was quantified and normalized to the purified recombinant wild-type enzyme using a reiterative process involving the Bradford assay and SDS-PAGE staining. Figure 2A shows a representative Coomassie blue staining of 1 µg of each protein, confirming accurate protein concentration determination and near homogeneity of each preparation. The gel analysis also indicates that the variant APE1 proteins exhibit similar migration properties to wild-type during electrophoresis (at an estimated molecular mass of ∼35 kDa), further supporting normal protein integrity.

Chemical denaturation experiments were used to determine whether the amino acid substitutions had an effect on the thermodynamic stability of protein folding. APE1 and the variants were incubated in buffer containing varying concentrations of GndHCl, and monitored for protein unfolding by tryptophan fluorescence. The wavelength of maximal fluorescence (λ_max_) increases as tryptophan residues become exposed to solvent upon protein denaturation. Thermodynamic parameters associated with folding stability were obtained by fitting the dependence of λ_max_ on [GdnHCl] to eq. 1 (see Materials and Methods), where Δ*G*_uw_ is the extrapolated free energy of protein unfolding in the absence of denaturant, and *m*_eq_ is the sensitivity of the protein to GdnHCl (Figure 2B,C). The analysis revealed that wild-type APE1 and the variants exhibit very similar denaturation profiles and thermodynamic parameters, indicating that the mutations do not substantially alter the overall stability of folding for APE1.

### Functional Characterization of APE1 Variant Proteins

#### AP endonuclease activity

**Figure 3:**
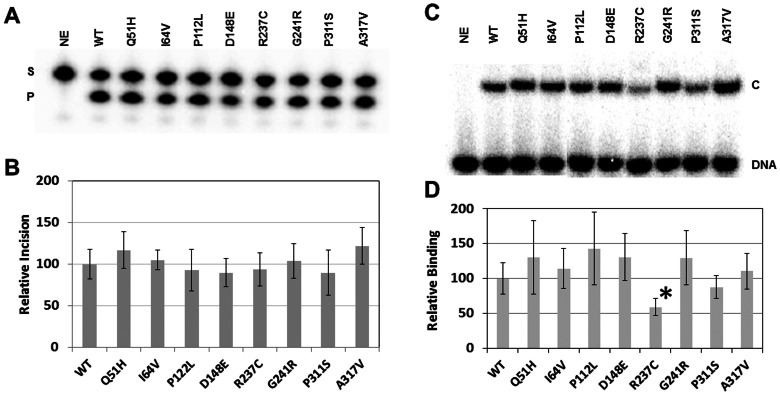
AP endonuclease incision and AP-DNA binding. (**A**) Wild-type (WT) and variant APE1 proteins (30 pg) were incubated with ^32^P-labeled 18F NMR DNA substrate (0.5 pmol), and the reactions were resolved on a urea-polyacrylamide denaturing gel. The non-incised substrate (S) and incision product (P) bands were visualized and quantified using standard phosphorimaging analysis. NE = no enzyme. (**B**) Relative AP site incision efficiency. Shown are the average and standard deviations of at least 6 independent reactions. (**C**) Wild-type (WT) and variant APE1 proteins (2 ng) were incubated with ^32^P-labeled 18F NMR DNA (100 fmol), and the binding reactions were resolved on a non-denaturing polyacrylamide gel. Standard phosphorimager analysis was employed to visualize unbound substrate (DNA) and the APE1-substrate complex (C). NE = no enzyme. (**D**) Relative AP-DNA binding affinity. Shown are the average and standard deviations of relative complex formation for at least 4 independent assays. *, p = 0.002.

After determining recombinant protein integrity and concentration, we measured the AP endonuclease activity of the different APE1 variants as compared to the wild-type protein. *In vitro* biochemical incision assays were performed using a ^32^P-labeled duplex DNA substrate that harbors a single abasic site analog residue, tetrahydrafuran (F), at a central position within one of the oligonucleotide strands (Figure 1B). After execution of the incision reactions, substrate and product were resolved on a denaturing gel (Figure 3A). From multiple independent experimental runs, it was determined that each APE1 variant displays an incision efficiency similar to that of the wild-type protein (designated as 100; Figure 3B).

To further examine the impact of the different amino acid substitutions on APE1 AP site repair function, we assessed AP-DNA binding activity using a previously developed, qualitative electrophoretic mobility shift assay (EMSA) [28]. The various recombinant proteins were incubated with the ^32^P-labeled abasic DNA substrate, in the absence of the catalytically-essential Mg^2+^ cofactor, and substrate binding was examined on a non-denaturing polyacrylamide gel (Figure 3C); the inclusion of EDTA in the binding reaction prevents DNA nicking by APE1 [28]. The relative affinity (% bound) of each variant was quantified compared with wild-type APE1, and except for R237C, all variants showed normal AP-DNA complex stability (Figure 3D). In the case of R237C, although it displayed wild-type endonuclease activity (see above), its DNA binding function was diminished ∼2-fold (p = 0.002). Figure S2 in File S1 shows that R237C exhibits a defect in DNA binding across a range of protein concentrations in comparison to wild-type APE1. Steady-state kinetic analysis of the AP endonuclease activity of R237C revealed a similar V_max_ (1.86 nM/min) and K_M_ (37.4 nM) as seen for the wild-type protein (V_max_, 2.75 nM/min; K_M_, 42.7 nM), suggesting that R237C likely exhibits reduced complex stability during the electrophoresis process.

#### 3′-Exonuclease/repair activity

**Figure 4:**
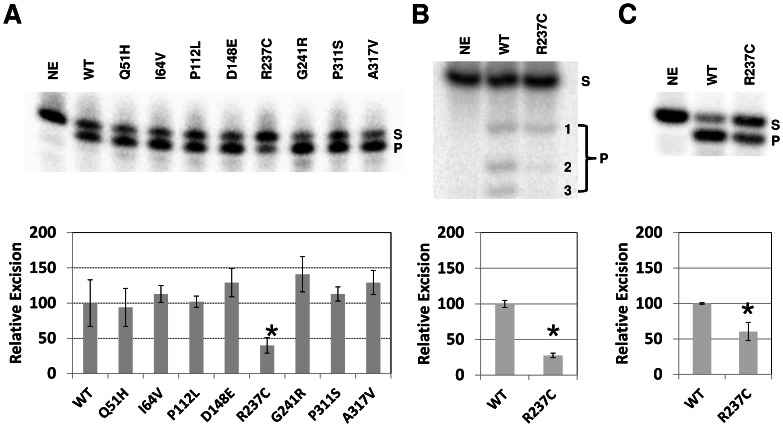
Exonuclease and 3′-repair assay. (**A**) Wild-type (WT) and variant APE1 proteins (250 ng) were incubated with ^32^P-labeled partially duplex 15P/34G DNA substrate (0.5 pmol), and the reactions were resolved on a high resolution denaturing sequencing gel (top). Substrate (S) and exonuclease degraded product (P) were visualized and quantified using standard phosphorimaging analysis. NE = no enzyme. Graph of relative 3′ to 5′ exonuclease efficiency is shown (bottom), depicting the average and standard deviations of 4 independent experiments. *, p = 0.01. (**B**) Partial duplex 17P/34G substrate (0.5 pmol) was incubated with APE1 proteins (250 ng) and products were resolved on a high resolution denaturing sequencing gel. Products were detected using phosphorimaging analysis. Densitometry results are graphed below from 3 independent assays. *, p = 0.0009. (**C**) The 3′-phosphate partial duplex 15-p/34G DNA substrate (0.5 pmol) was incubated with APE1 proteins (1 ng) and products were resolved on a high resolution denaturing sequencing gel. Densitometry results are graphed below from 3 independent assays. *, p = 0.01.

To gain a broader understanding of the impact of the amino acid variation on APE1 nuclease activities, we examined the 3′−5′exonuclease function of APE1. In the experiments here, we employed a 3′ recessed duplex substrate, comprised of a ^32^P-labeled 15 base oligonucleotide annealed to a complementary 34 base oligonucleotide (Figure 1C). As seen previously [14], upon electrophoresis of the enzyme reactions in a high resolution denaturing sequencing gel, the 15-mer showed degradation to a single major product band after incubation with the wild-type protein (Figure 4A, top image). While seven of the eight variants exhibited normal degradation activity, the R237C variant showed a significant ∼60% decrease in exonuclease function (p = 0.01) relative to the wild-type enzyme (Figure 4A, bottom graph). A similar ∼3-fold reduced 3′ to 5′ exonuclease activity (p = 0.0009) was observed for R237C on a 3′-recessed 17 base/34 base oligonucleotide duplex arrangement (Figure 4B, top image), with formation of the shorter products being most affected relative to the wild-type protein (Figure 4B, bottom graph). We also examined the ability of the R237C variant to process a 3′-damage-containing terminus, using a substrate identical to the 15P/34G configuration above, except with a phosphate group at the 3′-end of the 15 base oligonucleotide. As shown in Figure 4C, R237C degraded the labeled 15-p DNA strand at about half the efficiency of wild-type APE1 (p = 0.01).

#### Redox function

**Figure 5:**
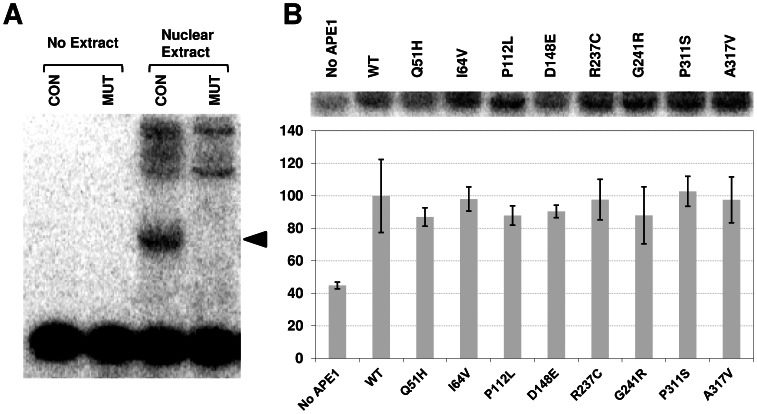
REF-1 assay. (**A**) HCT116 nuclear extract was incubated with ^32^P-labeled consensus (CON) or mutant (MUT) AP-1 oligonucleotide substrates, and binding reactions were resolved on a non-denaturing polyacrylamide gel. Control reactions without nuclear extract (no extract) are shown. The arrow designates the position of the AP-1-specific consensus binding complex, not seen with the MUT double-stranded DNA. Higher molecular weight non-specific complexes are observed. (**B**) Reduced wild-type (WT) or variant APE1 protein was incubated with HCT116 nuclear extract in the presence of the ^32^P-labeled AP-1 CON DNA substrate. Shown is the AP-1-specific complex in the absence (no protein) or presence of the indicated reduced APE1 protein after phosphorimager analysis. Plotted is the relative AP-1 DNA binding activity, in comparison with reduced WT protein. Values represent the average and standard deviation of 3 independent experimental points.

Separate from its roles in DNA processing, Curran and colleagues purified APE1 as a protein, which they termed redox-effector factor 1 (REF-1), that activates the DNA binding activity of the AP-1 transcription complex through a post-translational redox mechanism [16]. We measured the REF-1 function of the various APE1 proteins using a previously described methodology [29], in part because the Q51H and I64V substitutions (polymorphic variants) reside near the N-terminal region that is associated with redox activity (Figure 1A). In brief, reduced APE1 proteins were incubated with nuclear extracts that possess the c-Fos/c-Jun transcription factor complex (AP-1), and relative binding of ^32^P-labeled AP-1 target DNA (CON) was analyzed. As shown in Figure 5A, a clear AP-1 specific complex is observed in HCT116 nuclear extracts that is not seen in the absence of extract or with a mutant (MUT) ^32^P-labeled AP-1 DNA duplex. As expected, addition of reduced wild-type APE1 protein increased AP-1/DNA complex formation (Figure 5B, compare WT to “No APE1”). Notably, each of the purified APE1 variant proteins stimulated the AP-1 DNA binding activity of the HCT116 nuclear extract to a similar degree as seen with wild-type recombinant protein (Figure 5B).

### Activity of APE1 Variants in a Reconstituted Repair Assay

**Figure 6:**
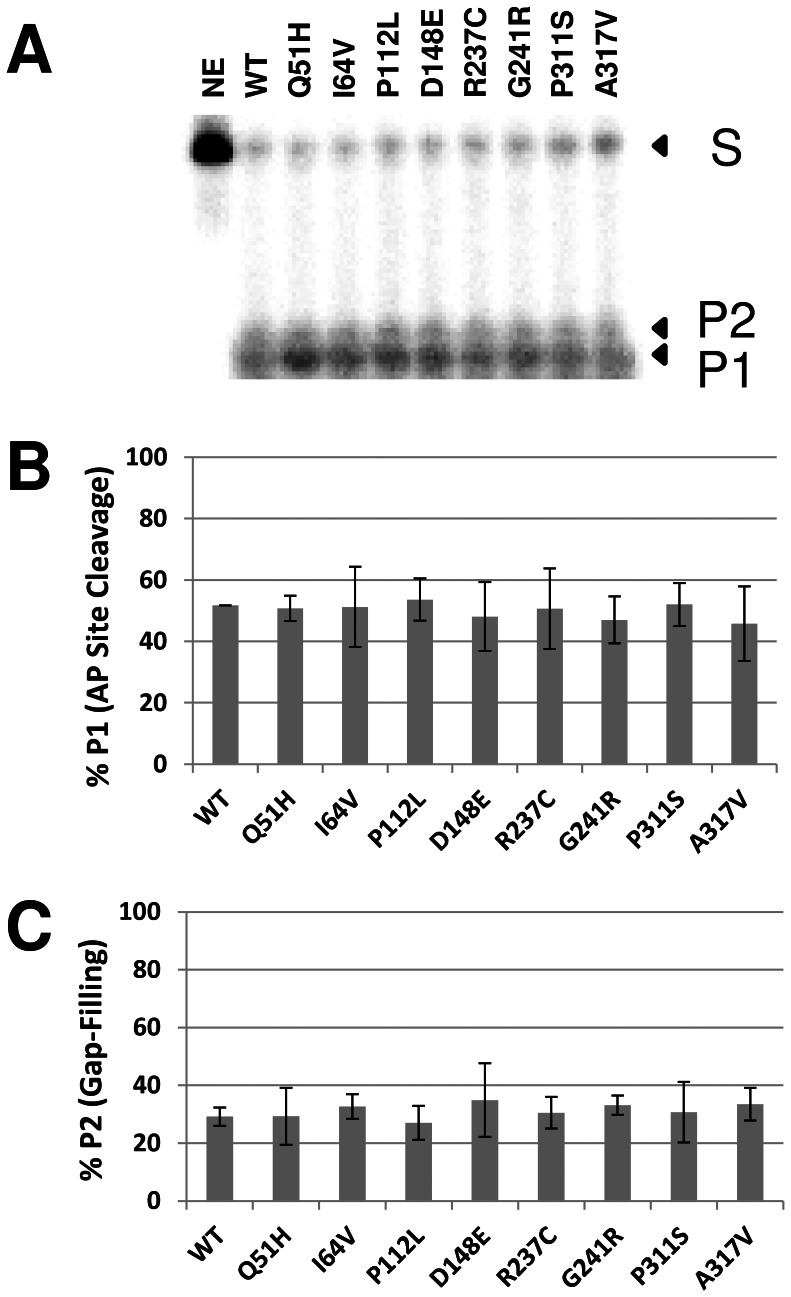
Reconstitution assay using purified BER proteins. (**A**) Wild-type (WT) and variant APE1 proteins were incubated with UDG and POLβ with ^32^P-labeled 34U DNA substrate (1 pmol), and the reactions were resolved on a urea-polyacrylamide denaturing sequencing gel. The non-incised substrate (S), AP site incision product (P1), and gap-filling extension product (P2) were visualized and quantified using standard phosphorimaging analysis. NE = no enzyme. (**B**) Relative AP site cleavage efficiency. Shown are the averages and standard deviations of 4 independent reactions. (**C**) Relative gap-filling activity. Shown are the average and standard deviation of 4 independent assays.

The enzymatic process of BER is thought to involve the coordinated recognition of a product/enzyme complex by the next protein in the pathway, in a so-called “passing-the-baton” mechanism [36]. As a means of assessing the effect of the different amino acid changes in APE1 on coordination during the first steps of BER, we incubated purified UDG, wild-type or variant APE1, and POLβ with a radiolabeled 34-mer uracil-containing oligonucleotide duplex (34U, Figure 1C). We monitored uracil excision, AP site cleavage and gap-filling, employing a protein ratio (∼0.5 UDG:1.0 APE1∶0.15 POLβ) that is consistent with previously measured cellular concentrations of the different human enzymes [37]. As shown in Figure 6, each APE1 variant reaction produced an overall AP site incision and gap-filling outcome similar to that of wild-type, consistent with the AP endonuclease results using the recombinant proteins alone (Figure 3A, B) and suggestive of normal interactions/coordination for the different variant proteins.

### Intracellular Localization of APE1 Variant Proteins

**Figure 7:**
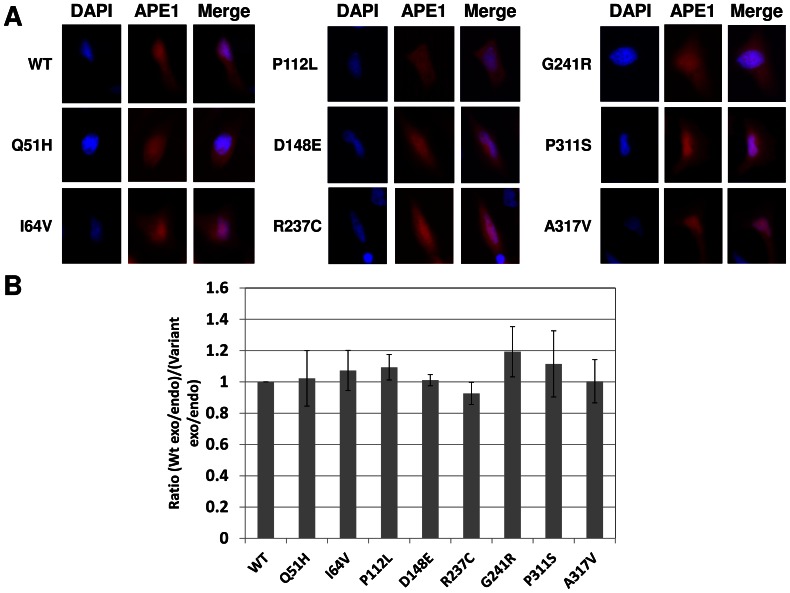
Intracellular localization of APE1 protein variants. (**A**) Representative microscopy images of the mCherry APE1 fusion proteins following plasmid transfection into HeLa cells. Shown are the DAPI nuclear staining, mCherry fusion protein fluorescence and the merged images. (**B**) Comparative cytoplasm to nuclear distribution for the different mCherry APE1 proteins. Using densitometry, the ratio of exogenous cytoplasmic mCherry-tagged wild-type (WT) APE1 protein to endogenous cytoplasmic protein was divided by the ratio of exogenous nuclear mCherry-tagged WT APE1 protein to endogenous nuclear protein, and this value was designated as 1. The identical ratio was then determined for each of the APE1 variant proteins, and plotted relative to the WT value. Shown is the average and standard deviation of results from 3 separate extract preparations and western blot experiments.

To assess for a potential biological effect of the amino acid change, we tagged each APE1 variant with an N-terminal mCherry fluorescent label and monitored intracellular localization after transfection of the different constructs into the HeLa human cervical cancer cell line by fluorescent microscopy. Figure 7A shows that wild-type mCherry-labeled APE1 (red) is most intensely concentrated in the nucleus (DAPI), although the protein localizes to the cytoplasm as well, presumably due to, at least in part, overexpression. Each of the variant APE1 proteins displayed a distribution pattern similar to that of wild-type (Figure 7A). To further evaluate intracellular localization of the different fusion proteins, nuclear and cytoplasmic protein extracts were prepared from HeLa cells after plasmid transfection. Extracts were then submitted to western blot analysis, and densitometry was performed on the endogenous and exogenous (mCherry-tagged) APE1 protein signals. The ratio of exogenous cytoplasmic protein to endogenous cytoplasmic protein was divided by the ratio of exogenous nuclear protein to endogenous nuclear protein, and plotted relative to wild-type APE1, which was arbitrarily designated as 1. As seen in Figure 7B, the results support the fluorescent imaging data and indicate that the APE1 variants exhibit a similar intracellular distribution as the wild-type protein.

### *APE1* Exon Variation in the NCI-60 Cancer Cell Line Panel

To more broadly examine APE1 variability in cancer, we re-sequenced the *APE1* exons of the 60 cancer cell lines within the NCI-60 panel, as well as in HeLa and glioblastoma T98G genomic DNA. Our efforts found no novel APE1 amino acid variants, yet revealed that almost half of the NCI-60 panel contained the polymorphic D148E variant; five of the 60 cell lines were homozygous E148 (Table 2). Interestingly, all renal cancer lines were wild-type for APE1, as was HeLa and T98G, and all skin cancers/melanomas were heterozygous D148/E148. The only missense mutation other than D148E (29.1% frequency) was the other common variant Q51H (0.008% frequency), observed in the colorectal cancer cell line HCT-15.

**Table 2:** Re-sequencing of *APE1* exons in the NCI-60 cancer cell line panel.

Cancer Type	Wild-Type (D/D)	Heterozygous (D/E)	Homozygous Variant (E/E)
**Leukemias (n = 6)**	1 (RPMI-8226)	3 (MOLT-4,CCRF-CEM, SR)	2 (K562, HL-60(TB))
**Brain (n = 6)**	5 (SF-268, SF-295, SF-539, SNB-75)	0	1 (U251)
**Breast (n = 6)**	3 (BT-549, MDA-MB-435, T-47D)	2 (HS-587T, DMA-MD-231/ATCC)	1 (MCF7)
**Colorectal (n = 7)**	6 (COLO 205, HCC-2998, HCT-15*, HT-29, KM12, SW-620)	1 (HCT-116)	0
**Lung (n = 10)**	4 (A549/ATCC, EKVX, HOP-62, MDA-MD-468)	6 (HOP-92, NCI-H322M, NCI-H226, NCI-H23, NCI-H460, NCI-H522)	0
**Skin/Melanomas (n = 8)**	0	8 (LOX IMVI, M14, SK-MEL-2, SK-MEL-28, SK-MEL-5, UACC-257, UACC-62, MALME-3M)	0
**Ovarian (n = 7)**	2 (OVACR-8, NCI/ADR-RES)	4 (IGR-OVR, OVCAR-3, OVCAR-5, SK-OV-3)	1 (OVCAR-4)
**Prostate (n = 2)**	1 (PC-3)	1 (DU-145)	0
**Renal (n = 8)**	8 (786-O, A498, ACHN, CAKI-1, RXF-393, SN12C, TK-10, UO-31)	0	0
**Total (n = 60)**	30	25	5

Re-sequencing was performed as described in Materials and Methods. All sequences were analyzed for homology to transcript variant 3 of APE1 (NM_080649.1). Genotypes have been divided into wild-type (D/D), heterozygous (D/E) or homozygous variant (E/E) for residue position 148 of APE1. Note: *, D/D for position 148, but Q/H heterozygous for position 51. Cancer type and cell line name are designated. Additional information is available at: http://dtp.nci.nih.gov/docs/misc/common_files/cell_list.html.

## Discussion

Deleterious mutations in the core BER participants, namely APE1, POLβ and XRCC1, have not been genetically linked to an inherited disorder. This apparent lack of BER “disease alleles”, which classically would involve a loss-of-function mutation, presumably stems from the essential role of the pathway in coping with the high rates of spontaneous, endogenous DNA damage. Consistent with this notion, biallelic null mutations in the core BER factors lead to early embryonic or post-natal lethality in mice [38]–[41]. As such, scientists have pursued the hypothesis that more mild reductions in BER capacity in humans, arising from more subtle variant “susceptibility alleles”, would associate with increased disease risk, most likely in an exposure- or lifestyle-influenced manner [5]. Through the work herein and conducted previously [33], [42], we have sought to explore the functional consequences of amino acid variation observed in core BER proteins, with the idea that non-conservative substitutions will give rise to reduced-function susceptibility alleles.

An important message from the studies within is that the different computational methods were often contradictory in terms of the predicted impact on protein structure/function and did not provide conclusive insights into the actual experimental outcomes. As one example, the Q51H variant was predicted to have a negative outcome in all three computational approaches, yet was observed to have no significant biochemical or structural defect. This observation stresses the importance of conducting experimental assessment of the functional consequence of an amino acid substitution. Towards this goal, we found that none of the population variants, *i.e.*, those not specifically associated with human disease (*e.g.*, Q51H, I64V, D148E, G241R, P311S, and A317V), exhibited altered function. Such a result may indicate that any impairment in APE1 activity will be incompatible with normal survival and thus not be preserved in the healthy population, or that the protein maintains a generally adaptable primary sequence composition. The fact that the polymorphic variants, Q51H, I64V or D148E (as seen in other efforts [33], [43]), had no functional or structural consequence suggests that these proteins do not play a direct role in disease risk, as has been promoted by at least a few epidemiological association studies [44]. Additionally, the cancer-associated P112L mutant behaved like wild-type in all assays, implying that this variant is not involved in the carcinogenic outcome. While the assay set employed within is by no means all-inclusive, given that most variants were similar to wild-type in a range of biochemical, biological and structural experiments, including single protein and simple reconstituted assays, there is no compelling reason to believe that they will be defective for other functional end-points.

The one variant that did display a defect was the endometrial cancer-associated protein R237C, which exhibited deficient 3′ to 5′ exonuclease and 3′-damage excision activities, as well as slightly reduced AP-DNA complex stability. Seemingly consistent with this observation, our prior work found that substitution of R237 with A resulted in an ∼2-fold reduced AP endonuclease function [33]. The R to A amino acid change gave rise to an apparent protein structural instability (based on reduced solubility, altered chromatographic properties, and freeze-thaw sensitivity), which was predicted to stem from disruption of the hydrogen bonding network between R237 and two negatively charged residues, E216 and E217. Although the precise mechanism for the impaired function of R237C is unknown, it seems reasonable to predict that like R237A (but apparently to a lesser extent), disturbance of the integrity of the above hydrogen bonding circuitry, which is positioned within α-helix number 9 of APE1 (a region of the protein that lies adjacent to the DNA binding groove), would adversely affect local structural integrity and thus overall complex stability with nucleic acid substrates. While R237C would appear to represent a reduced-function (inherited or somatic) susceptibility allele, which is defective in some aspect of autonomous exonuclease proofreading and 3′-damage removal that results in increased mutagenesis or genetic instability, such a hypothesis requires conformation using isogenic wild-type and mutant human cell lines or compatible genetically-engineered mouse models.

In light of our results with R237C, and since prior work suggested an association between amyotrophic lateral sclerosis (a.k.a., Lou Gehrig’s Disease) and reduced-function APE1 variants (*e.g.*, L104R,, E126D and D283G) [33], we searched for disease-associated variation in *APE1* in 62 human cancer cell lines. Surprisingly, particularly given the evidence of enormous sequence heterogeneity in cancer cells [45], which likely exhibit an intrinsic mutator phenotype [46], we found no novel APE1 amino acid sequence variants in the NCI-60 cancer cell line panel, or HeLa or T98G cancer cells. Thus, while inherited, typically loss-of-function mutations in BER-related genes are associated with human disease – such as MutY homolog (MYH)-associated polyposis (MAP), a hereditary colorectal cancer syndrome stemming from a defect in the MYH DNA glycosylase; hyper-IgM syndrome V (HIGMV), an immunodeficiency disorder resulting from a defect in uracil DNA glycosylase; and the neurological diseases, spinocerebellar ataxia with axonal neuropathy (SCAN1), ataxia with oculomotor apraxia 1 (AOA1), and microcephaly, early-onset, intractable seizures and developmental delay (denoted MCSZ), which arise from ineffective single-strand break repair – it is noteworthy that few reduced-function variants have been observed among the core BER factors [5]. Indeed, in our previous efforts exploring several XRCC1 variants, only R280H was found to display significantly altered activity, although the functional capacity of this and other XRCC1 variants remains unclear ([42] and references therein). Two major challenges for the BER community going forward will be to more broadly determine whether there exist inheritable reduced-function alleles in the core BER components that give rise to disease susceptibility or whether somatic BER mutations arise that are causative in disease etiology, such as appears to be the case for POLβ [47]–[49].

## Supporting Information

File S1 **Figure S1 & S2 and Table S1 & S2.**
(PDF)
