# Supplementary material for: Functional Assessment of Population and Tumor-Associated APE1 Protein Variants
Source: PLoS One. 2013 Jun 11;8(6):e65922. doi: 10.1371/journal.pone.0065922 (PMC3679070; doi:10.1371/journal.pone.0065922)
Supplement: File S1 — Figure S1 & S2 and Table S1 & S2. (PDF) [file pone.0065922.s001.pdf]

Figure S1

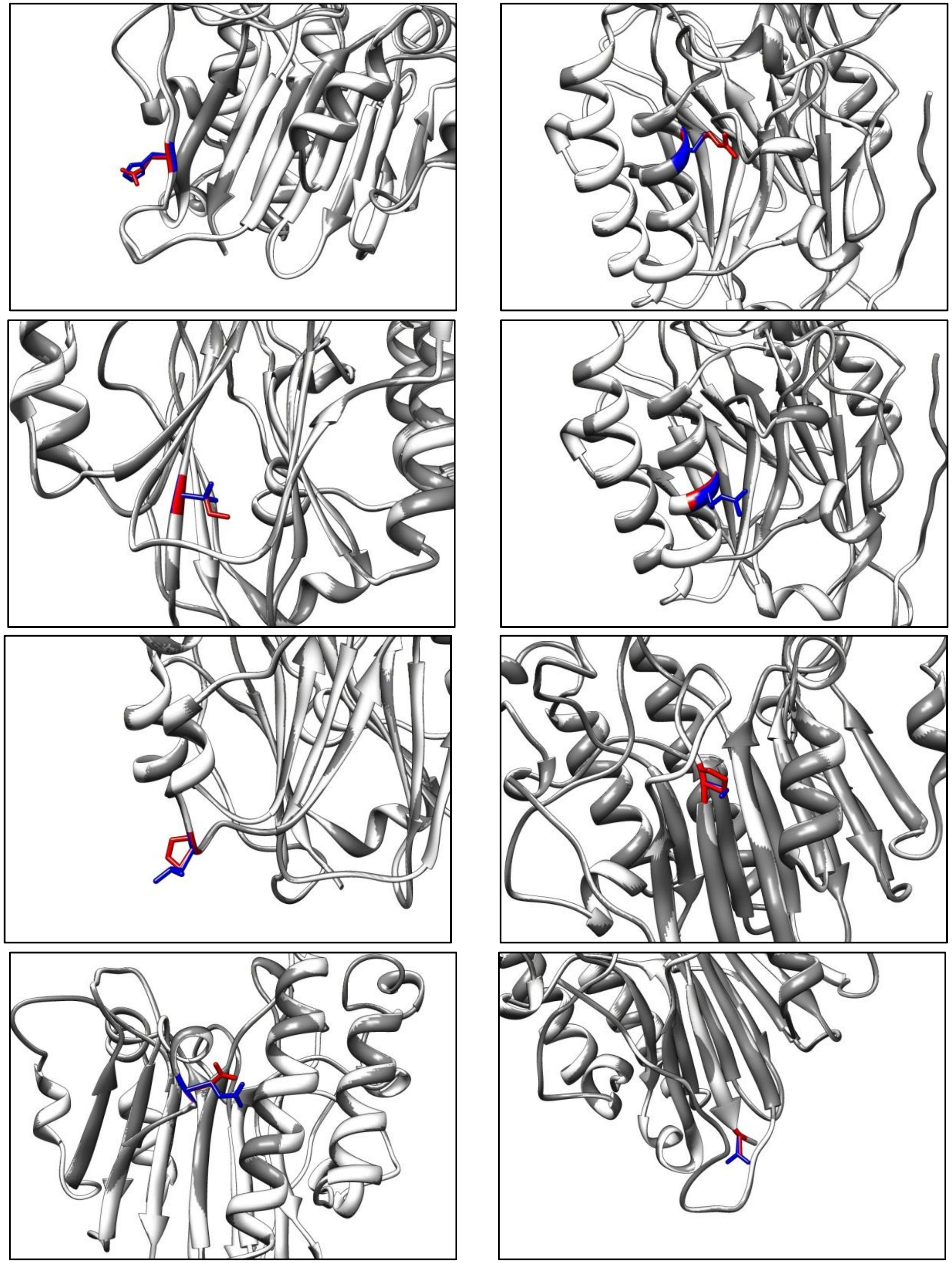

Q51H

R237C

I64V

G241R

P112L

P311S

D148E

A317V

Figure S1. Molecular modeling of APE1 protein variants. The wild-type APE1 amino acid sequence was obtained (NCBI accession# AF488551) and changed as per the mutations examined here (Q51H, I64V, P112L, D148E, R237C, G241R, P311S, and A317V). The PDB structure of wild-type APE1 was PDB ID# 1DE8, and the variant models were generated using SWISS-MODEL PDB2PQR (http://pdb2pqr.sourceforge.net/). The modeling was analyzed in UCSF Chimera software by overlaying the wild-type amino acids (white backbone, red amino acid) with the variant residue (grey backbone, blue amino acid) to compare alterations in protein integrity.

Figure S2

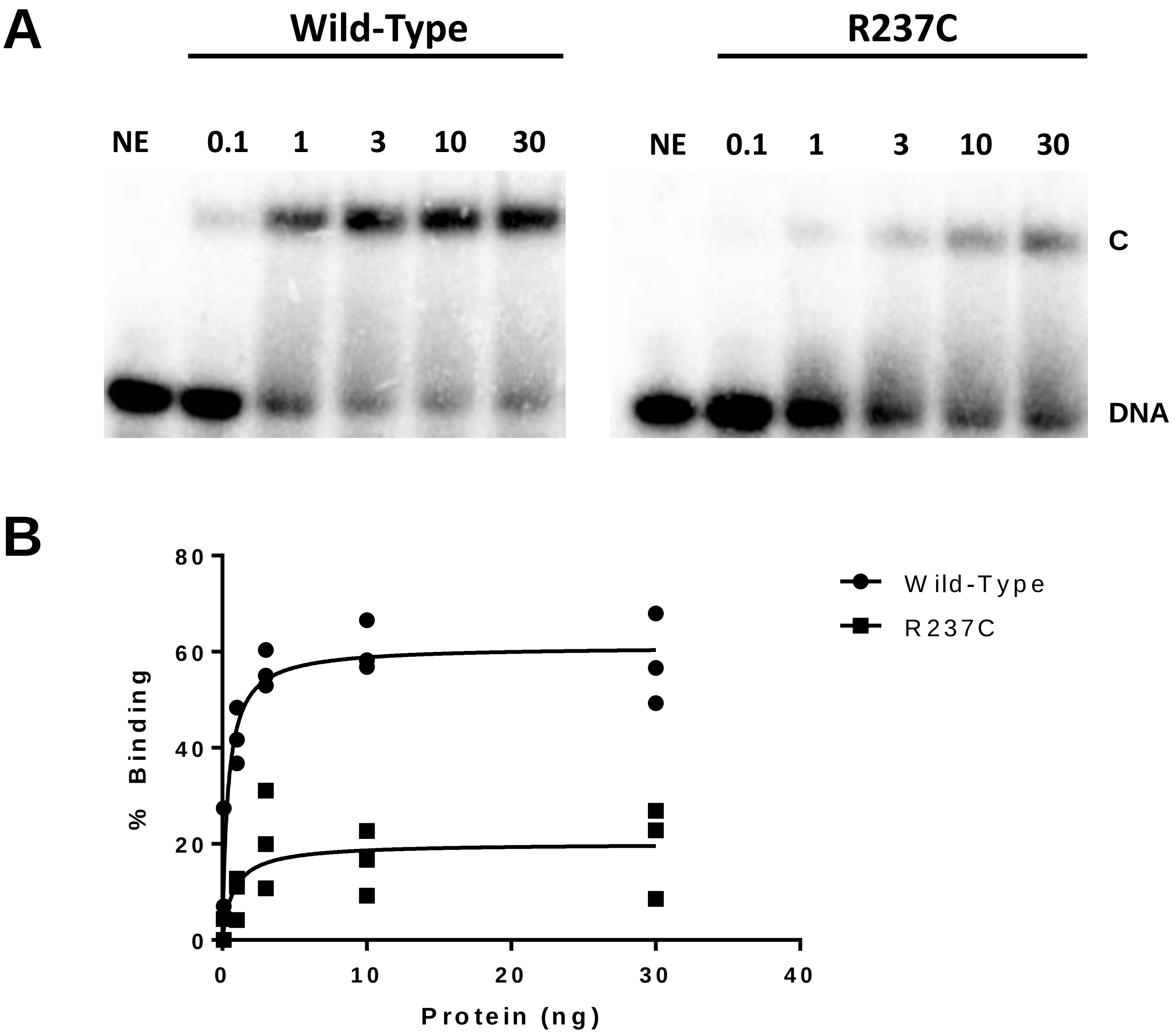
A
Wild-Type
R237C
NE
0.1
1
3
10
30
C
DNA
B
% Binding
Protein (ng)
Wild-Type
R237C
0
20
40
60
80
10
30

Figure S2. AP-DNA binding of wild-type and R237C APE1 proteins. (A) Wild-type or R237C variant protein (0.1 to 30 ng, as designated) were incubated with ^{32}P-labeled 18F NMR DNA (100 fmol), and the binding reactions were resolved on a non-denaturing polyacrylamide gel as described in Materials and Methods. Standard phosphorimager analysis was employed to visualize unbound substrate (DNA) and the APE1-substrate complex (C), with a representative gel image shown. NE = no enzyme. **(B)** Plot of the three independent data points for each protein at the indicated protein concentration. The line of best fit is shown.

Table S1. Variant primer set for the indicated amino acid change and each oligonucleotide name and sequence. The mutated codon is underlined.

Change	Oligonucleotide name and sequence (5'-3'):	
Q51H	Q51H-For:	GAC CCC CCA GAT CAC AAA ACC TCA CCC AG
	Q51H-Rev:	CTG GGT GAG GTT TTG TGA TCT GGG GGG TC
I64V	I64V-For:	AAC CTG CCA CAC TCA AGG TCT GCT CTT GGA ATG TG
	I64V-Rev:	CAC ATT CCA AGA GCA GAC CTT GAG TGT GGC AGG TT
P112L	P112L-For:	TGA ACT TCA GGA GCT GCT TGG ACT CTC TCA TCA AT
	P112L-Rev:	ATT GAT GAG AGA GTC CAA GCA GCT CCT GAA GTT CA
D148E	D148E-For:	CTT ACG GCA TAG GCG AGG AGG AGC ATG ATC AGG
	D148E-Rev:	CCT GAT CAT GCT CCT CCT CGC CTA TGC CGT AAG
R237C	R237C-For:	CAC GCC ACA AGA GTG CCA AGG CTT CGG
	R237C-Rev:	CCG AAG CCT TGG CAC TCT TGT GGC GTG
G241R	G241R-For:	GAG CGC CAA GGC TTC AGG GAA TTA CTG CAG G
	G241R-Rev:	CCT GCA GTA ATT CCC TGA AGC CTT GGC GCT C
P311S	P311S-For:	TCG GCA GTG ATC ACT GTT CTA TCA CCC TAT ACC TA
	P311S-Rev:	TAG GTA TAG GGT GAT AGA ACA GTG ATC ACT GCC GA
A317V	A317V-For:	ATC ACC CTA TAC CTA GTA CTG TGA GGA TCC GGC
	A317V-Rev:	GCC GGA TCC TCA CAG TAC TAG GTA TAG GGT GAT

Table S2. Amplification primers used in *APE1* exonic sequencing. The underlined portion is the region complementary to the APE1 genomic sequence. The remaining portion of the oligonucleotide is the binding site for M13 forward and reverse primers used in direct sequencing of the PCR product.

Exon	Oligonucleotide sequence (5'-3')
Exon 1&2	Fex1_2: GTTTTCCCAGTCACGAC<u>GAGGCTAAGCGTCTCCGTCAC</u>
	Rex1_2: AGGAAACAGCTATGACCAT<u>CTGCGACTTCTTCACAAACC</u>
Exon 3	Fex3: GTTTTCCCAGTCACGAC<u>GGTTTGTGAAGAAGTCGCAG</u>
	Rex3: AGGAAACAGCTATGACCA<u>TCCTGAAGGCTAAACGGAGAA</u>
Exon 4	Fex4: GTTTTCCCAGTCACGAC<u>GAATATTGTGCTGCTTGACTC</u>
	Rex4: AGGAAACAGCTATGACCAT<u>GGGAAAGCAATCAAGAGGTG</u>
Exon 5	Fex5: GTTTTCCCAGTCACGAC<u>TTGCTAATTCTCTATCTCTG</u>
	Rex5: AGGAAACAGCTATGACCAT<u>GAGTGTTTAAAGAAGGAATGG</u>
